# Exogenous Nucleotides Ameliorate Insulin Resistance Induced by Palmitic Acid in HepG2 Cells through the IRS-1/AKT/FOXO1 Pathways

**DOI:** 10.3390/nu16121801

**Published:** 2024-06-07

**Authors:** Lixia Song, Yong Li, Meihong Xu

**Affiliations:** 1 Department of Nutrition and Food Hygiene, School of Public Health, Peking University, Beijing 100191, China; slx18219898353@163.com (L.S.); liyong@bjmu.edu.cn (Y.L.); 2 Beijing Key Laboratory of Toxicological Research and Risk Assessment for Food Safety, Peking University, Beijing 100191, China; 3 Institute of Medical Technology, Peking University Health Science Center, Beijing 100019, China

**Keywords:** exogenous nucleotides, HepG2 cell, insulin resistance

## Abstract

Nucleotides (NTs) act as pivotal regulatory factors in numerous biological processes, playing indispensable roles in growth, development, and metabolism across organisms. This study delves into the effects of exogenous NTs on hepatic insulin resistance using palmitic-acid-induced HepG2 cells, administering interventions at three distinct dosage levels of exogenous NTs. The findings underscore that exogenous NT intervention augments glucose consumption in HepG2 cells, modulates the expression of glycogen-synthesis-related enzymes (glycogen synthase kinase 3β and glycogen synthase), and influences glycogen content. Additionally, it governs the expression levels of hepatic enzymes (hexokinase, phosphoenolpyruvate carboxykinase, and glucose-6-phosphatase). Moreover, exogenous NT intervention orchestrates insulin signaling pathway (insulin receptor substrate-1, protein kinase B, and forkhead box protein O1) and AMP-activated protein kinase (AMPK) activity in HepG2 cells. Furthermore, exogenous NT intervention fine-tunes the expression levels of oxidative stress-related markers (malondialdehyde, glutathione peroxidase, and NADPH oxidase 4) and the expression of inflammation-related nuclear transcription factor (NF-κB). Lastly, exogenous NT intervention regulates the expression levels of glucose transporter proteins (GLUTs). Consequently, exogenous NTs ameliorate insulin resistance in HepG2 cells by modulating the IRS-1/AKT/FOXO1 pathways and regulate glucose consumption, glycogen content, insulin signaling pathways, AMPK activity, oxidative stress, and inflammatory status.

## 1. Introduction

Insulin resistance (IR) is characterized by a notable decline in insulin’s efficacy due to functional impairments in the insulin receptor or its downstream signaling molecules [1]. It serves as an early determinant in the pathogenesis of type 2 diabetes (T2DM) and poses a substantial risk for comorbidities such as hypertension, dyslipidemia, and cardiovascular ailments, rendering it a focal point in endocrine metabolism research. Principal targets of insulin action encompass the liver, adipose tissue, and skeletal muscles. The liver, pivotal in glucose and lipid metabolism, orchestrates blood glucose homeostasis via processes like glycogen synthesis, glycogenolysis, and gluconeogenesis. Decreased hepatic insulin sensitivity precipitates insulin resistance. Various models, including inflammation, oxidative stress, and ectopic lipid deposition, have been posited to elucidate the mechanisms underpinning insulin resistance [2,3].

Given the deleterious impact of insulin resistance on physiological functions, preserving and enhancing insulin sensitivity holds paramount importance for optimizing overall health. The pursuit of safe and efficacious intervention strategies is of utmost significance. In recent years, emerging lifestyle interventions such as aerobic exercise and resistance training have demonstrated promise in mitigating insulin resistance among populations afflicted with metabolic disorders. However, these interventions exhibit limited applicability, particularly among elderly individuals with a higher prevalence of underlying conditions. Conversely, nutritional interventions possess attributes such as minimal side effects, sustained efficacy, broad applicability, and cost-effectiveness, thereby assuming a pivotal role. Presently, nutritional interventions targeting insulin resistance center on dietary patterns [4,5,6,7,8], plant-derived compounds [9,10,11,12,13], probiotics [14,15,16], and other modalities. Nevertheless, many of these compounds predominantly exert singular effects.

Nucleotides (NTs) constitute the fundamental constituents of nucleic acids, comprising nitrogenous bases, pentose sugars, and phosphate groups. In the human body, nucleotides serve as the molecular backbone of nucleic acids, crucial for genetic information transmission. Moreover, they play pivotal roles in regulating diverse metabolic processes as either free nucleotides or their derivatives, functioning as chemical energy carriers, signaling molecules, and constituents of coenzymes [17]. Found in breast milk and daily dietary intake, studies spanning multiple generations and lifelong consumption have underscored their safety and enduring preventive utility. Notably, nucleotides serve as vital additives in infant formula milk. Categorized into endogenous and exogenous types, endogenous nucleotides represent the principal source within the human organism. Nucleotide synthesis occurs via de novo synthesis and salvage synthesis pathways, with de novo synthesis being the primary route under normal physiological conditions. However, during certain abnormal circumstances where de novo synthesis fails to meet the demands of heightened cellular metabolism, supplementation with nucleotide supplements becomes imperative for salvage synthesis. Research has demonstrated the multifaceted biological functions of exogenous NTs, encompassing immune modulation [18], promotion of infant growth and development [19], protection of gastrointestinal [20,21] and hepatic function [22], enhancement of memory [23], anti-inflammatory [24] and antioxidant effects [25,26], regulation of lipid metabolism [27], and improvement in muscle cell insulin resistance [28]. Notably, insulin resistance is a significant risk factor for cardiovascular diseases, highlighting the necessity for effective interventions. Exogenous nucleotides present promising advantages, such as immune modulation, hepatic protection, and lipid metabolism regulation. Existing studies indicate their potential in ameliorating muscle cell insulin resistance and modulating insulin sensitivity through diverse pathways. However, there is currently limited research on their specific intervention effects concerning hepatic insulin resistance. 

Hence, this study employed in vitro cell experiments to construct a palmitic acid (PA)-induced in vitro hepatic insulin-resistant HepG2 cell model. The objective was to investigate the impacts and mechanisms of exogenous nucleotides on hepatic insulin resistance at the cellular level, thereby laying a theoretical groundwork for enhancing insulin sensitivity.

## 2. Materials and Methods

### 2.1. Test Substance

The exogenous NT mixture (AMP/CMP/GMP/UMP = 16:41:19:24) is a white solid powder provided by Dalian Zhenao Biotechnology Co., Ltd. (Dalian, China) [21,24].

### 2.2. Main Reagents and Instruments

#### 2.2.1. Main Reagents

The main reagents and instruments were as follows: DMEM high-glucose culture medium (GIBCO, Grand Island, NE, USA), fetal bovine serum (GIBCO, Grand Island, NE, USA), streptomycin mix solution (100×) (Coolaber, Beijing, China), palmitic acid/stearic acid (Coolaber, Beijing, China), bovine serum albumin (fatty acid-free) (Yuan Ye, Shanghai, China), recombinant human insulin solution (Pricella, Wuhan, China), phosphate buffer solution (Hyclone, Shanghai, China), CCK-8 cell proliferation assay kit (KeyGEN, Shanghai, China), GSH-PX assay kit (Nanjing Jiancheng Bioengineering Institute, Nanjing, China), LDH assay kit (Nanjing Jiancheng Bioengineering Institute, Nanjing, China), MDA assay kit (Nanjing Jiancheng Bioengineering Institute, Nanjing, China), PEPCK assay kit (Nanjing Jiancheng Bioengineering Institute, Nanjing, China), an HK assay kit (Nanjing Jiancheng Bioengineering Institute, Nanjing, China), glycogen assay kit (Nanjing Jiancheng Bioengineering Institute, Nanjing, China), BCA protein quantitative detection kit (ready to use) (UElandy, Suzhou, China), protein extraction reagent (Tian Derui, Beijing, China), 10×tris-glycine-SDS electrophoresis buffer (Tian Derui, Beijing, China), 10×TBST (pH8.0) (Tian Derui, Beijing, China), Rainbow 180 broad spectrum protein marker (11-180KD) (Tian Derui, Beijing, China), NC membrane (0.45 μm pore size) (Millipore, Burlington, MA, USA), PMSF (Amresco, Wayne, NJ, USA), acrylamide (Amresco, Wayne, NJ, USA), bis-acrylamide (Amresco, Wayne, NJ, USA), APS (Amresco, Wayne, NJ, USA), ECL (Millipore, Burlington, MA, USA).

#### 2.2.2. Main Instruments

The main instruments were as follows: a carbon dioxide incubator (MC0-15AC, Sanyo Electric Co., Ltd., Osaka, Japan), multifunctional microplate reader (BMG FLUOstar Omega, BMGLABTECH GmbH, Offenburg, Germany), high-speed refrigerated centrifuge (Eppendorf, Eppendorf AG, Hamburg, Germany), electronic balance (Sartouris, Gottingen, Germany), Mini P-4 electrophoresis tank (Cavoy, Beijing, China), wet transfer electrophoresis tank (Cavoy, Beijing, China), electrophoresis apparatus (Bio-Rad, Hercules, USA), shaking incubator (Thermo Fisher Scientific, Waltham, MA, USA), pH meter (Sartorius, Gottingen, Germany), and homogenizer (Monad, Cambridge, MA, USA ).

### 2.3. Experimental Cells and Treatmentss

HepG2 cells from a human hepatocellular carcinoma cell line were procured from the Cell Bank of the Chinese Academy of Sciences in Shanghai, China. These cells exhibited an adherent phenotype and were routinely cultured in DMEM medium supplemented with 10% fetal bovine serum and 1% penicillin–streptomycin at 37 °C in a humidified atmosphere with 5% CO_2_. Passage of cells occurred every 3–4 days upon reaching 80–90% confluence through trypsin digestion, followed by subculturing. Experimental procedures were conducted utilizing cells in the logarithmic growth phase, ensuring optimal growth conditions.

#### 2.3.1. Preparation of PA Solution

First, a 10% BSA solution was prepared by weighing out 0.8 g and adding it to 8 mL of distilled water, followed by complete dissolution in a water bath at 55 °C. Subsequently, 800 μL of a 300 mM PA solution dissolved in anhydrous ethanol was slowly added to the 10% BSA solution while stirring thoroughly until complete dissolution occurred in the 55 °C water bath. This yielded a 27 mM PA stock solution. The solution was sterilized using a 0.22 micrometer filter membrane and aliquoted and stored frozen.

#### 2.3.2. Establishment of the HepG2-IR Cell Model

After reaching confluence in adherent culture, HepG2 cells in the logarithmic growth phase were selected. Following recommendations from the relevant literature, various concentrations of palmitic acid (PA) were employed for intervention to establish an insulin-resistant cell model. The concentrations tested included 0, 0.10, 0.15, 0.20, 0.25, and 0.30 mM. Following a 24 h incubation period, glucose levels in the supernatant of the culture medium were assessed using a glucose assay kit (Acmec, Shanghai, China) to evaluate glucose consumption by the cells in each group. Subsequently, cell viability was evaluated at each concentration. The optimal insulin resistance model for HepG2 cells was determined based on glucose consumption and cell viability assessments. In this study, a PA concentration of 0.25 mM was selected for further experiments.

#### 2.3.3. Cell Groups

The cells were divided into six groups as follows:

Control Group (Control): HepG2 cells in optimal growth conditions were cultured in complete normal medium, and equivalent BSA solution concentrations were maintained as the control group.

Insulin-Resistant Model Group (IR-HepG2): HepG2 cells in optimal growth conditions were cultured in complete medium supplemented with 5% FBS and 0.25 mM PA for 24 h. Subsequently, during the intervention phase, they were maintained in the same medium for an additional 24 h.

Low Dose of NTs Group (NTs-L): HepG2 cells in optimal growth conditions were cultured in complete medium supplemented with 5% FBS and 0.25 mM PA for 24 h. Following this, during the intervention phase, they were cultured in complete medium containing 5% FBS, along with 50 μmol/L NTs and 0.25 mM PA, for an additional 24 h.

Medium Dose of NTs Group (NTs-M): HepG2 cells in optimal growth conditions were cultured in complete medium supplemented with 5% FBS and 0.25 mM PA for 24 h. During the intervention phase, they were cultured in complete medium containing 5% FBS, along with 100 μmol/L NTs and 0.25 mM PA, for an additional 24 h.

High Dose of NTs Group (NTs-H): HepG2 cells in optimal growth conditions were cultured in complete medium supplemented with 5% FBS and 0.25 mM PA for 24 h. Subsequently, during the intervention phase, they were cultured in complete medium containing 5% FBS, along with 200 μmol/L NTs and 0.25 mM PA, for an additional 24 h.

Positive Control Group (Met): HepG2 cells in optimal growth conditions were cultured in complete medium supplemented with 5% FBS and 0.25 mM PA for 24 h. During the intervention phase, they were cultured in complete medium containing 5% FBS, along with 0.1 mg/mL metformin and 0.25 mM PA, for an additional 24 h.

Following the intervention phase, insulin treatment was administered at a concentration of 1 × 10^−6^ mol/L for 40 min. Cells and supernatants were then collected for relevant indicator measurements.

### 2.4. The Experimental Method

#### 2.4.1. Cell Viability Assay

Cells were plated at a density of 100 μL/cell per well, corresponding to approximately 1 × 10^4^ cells, in a 96-well cell culture plate. Following treatment according to experimental protocols, 10 μL of CCK-8 detection solution was added to each well and then incubated at 37 °C for 1–4 h. Subsequently, the absorbance at a wavelength of 450 nm was measured using an enzyme-linked immunosorbent assay (ELISA) reader for each well.

#### 2.4.2. Glucose Consumption Measurement

Cells (approximately 1 × 10^4^ cells) were cultured at a volume of 100 μL per well in a 96-well cell culture plate. Following treatment with various experimental groups, glucose production in each cell group was assessed using the guidelines provided by the glucose assay kit. A standard curve was generated, and glucose concentration was determined utilizing the formula: Glucose concentration (mmol/L) = Standard concentration × (Sample well OD − Blank well OD)/(Standard well OD − Blank well OD). Glucose consumption (mmol/L) was calculated as the difference between glucose content in the blank well and the measurement well, with normalization to 1 for the control group.

#### 2.4.3. Glycogen Content Determination

Cells were seeded at a density of 3 × 10^5^ cells per well in a 6-well plate. Following treatment with various experimental conditions, the cells underwent three washes with PBS. Subsequently, cell collection was conducted for glycogen detection as per the manufacturer’s guidelines for the glycogen assay kit. Additionally, the protein content of each HepG2 cell group was quantified using the BCA method. The glycogen-to-protein ratio (mg/mg) was calculated and normalized to 1 for the control group.

#### 2.4.4. Liver Enzyme Assay

The cells were initially seeded at a density of 3 × 10^5^ cells per well in a 6-well plate. Following treatment with different experimental conditions, the cells underwent three washes with PBS. Subsequently, cell samples were collected to assess PEPCK and HK activities using the PEPCK assay kit and hexokinase (HK) activity assay kit, respectively, following the manufacturer’s instructions. Additionally, the protein content of each HepG2 cell group was quantified using the BCA method. The ratio of PEPCK or HK activity to total protein (mg) was then calculated, with normalization to 1 for the control group.

#### 2.4.5. Oxidative Stress Biomarker Detection

The cells were seeded at a density of 3 × 10^5^ cells per well in a 6-well plate. Following treatment with various experimental conditions, the cells underwent three washes with PBS. Subsequently, cell collection was conducted for LDH, MDA and GSH-PX detection utilizing assay kits according to the manufacturer’s instructions. Additionally, the protein content of each group of HepG2 cells was quantified using the BCA method. The ratio of MDA or GSH-Px to total protein (mg) was calculated, with normalization to 1 for the control group.

#### 2.4.6. Western Blot

The cells were cultured in a 6-well cell culture plate and treated with different experimental groups, each with 3 replicates. Subsequently, the cells were digested using trypsin, and the supernatant was discarded after centrifugation at 1000 rpm for 5 min. The cells were then washed twice with pre-chilled PBS on ice before storing the cell pellet at -80 °C. Western blot analysis was performed to detect various proteins, including AMPK (1:1000), p-AMPK (Thr172) (1:1000), AKT (1:2000), p-AKT (Ser473) (1:1000), IRS-1 (1:1000), p-IRS-1 (Ser307) (1:500), FOXO1 (1:1000), p-FOXO1 (Ser256) (1:1000), GS (1:1000), p-GS (Ser641) (1:1000), GSK3β (1:1000), p-GSK3β (Ser9) (1:1000), G6pase (1:1000), GLUT2 (1:1000), GLUT4 (1:1000), NOX4 (1:500), and NF-κB (1:2000).

(1) Tissue protein extraction was performed by employing pre-chilled RIPA protein extraction reagent, augmented with a protease inhibitor cocktail and a phosphatase inhibitor specifically targeting phosphorylated proteins. Prior to extraction, 0.1M PMSF stock solution was added to achieve a final concentration of 1 mM PMSF. Tissue samples were homogenized in lysis buffer at a 1:9 (weight/volume) ratio using a Fluka electric tissue homogenizer at 15,000 rpm for three cycles of 10 s with 10 s intervals, while maintaining low temperature in an ice/water mixture. Following homogenization, the samples underwent a 20 min incubation on ice, followed by centrifugation at 13,000 rpm for 20 min at 4 °C. Subsequently, the resultant supernatant was gathered, divided into aliquots, and stored appropriately. Then, protein concentration was quantified using the BCA method, and adjustments were made by diluting with RIPA buffer and incorporating 5× reducing sample buffer to achieve a final concentration of 2 mg/mL, followed by protein denaturation through boiling for 5 min.
(2) Western blot: Either 12% or 8% separation gels were prepared based on the target protein’s molecular weight, incorporating a 5% stacking gel. A total of 20 μg of protein was loaded per well. Electrophoresis conditions entailed applying a constant 90 V for approximately 20 min to the stacking gel and 160 V to the separation gel, with endpoint determination using pre-stained protein markers. Wet transfer was performed at a constant current of 300 mA using a 0.45 μm pore size NC membrane, transferring for 1 h for 12% separation gels or 2 h for 8% separation gels. Post-transfer, the membrane was stained with Ponceau S solution to evaluate transfer efficiency and mark the lanes. The membrane was blocked in 3% BSA-TBST at room temperature for 30 min with gentle shaking. The primary antibody was diluted in 3% BSA-TBST, incubating initially at room temperature for 10 min, followed by overnight incubation at 4 °C. The next day, the membrane was equilibrated to room temperature and incubated for an additional 30 min. The membrane was washed with TBST five times for 3 min each. The secondary antibody, goat anti-rabbit IgG (H+L) HRP, was diluted in 5% skim milk-TBST at a ratio of 1:10,000 and shaken gently at room temperature for 40 min. Subsequently, the membrane was washed with TBST six times for 3 min each. Finally, visualization was carried out using ECL chemiluminescence detection reagent, and grayscale intensity was analyzed using Image-J software (Total Lab Quant V11.5, Newcastle upon, Tyne, UK).

### 2.5. Statistical Analysis

The experimental results were presented as mean ± standard deviation. Data analysis was performed using SPSS 26.0 software. Initially, the data were subjected to homogeneity of variance analysis. If the data met the assumption of homogeneity of variance, one-way ANOVA was applied. For non-normally distributed or heteroscedastic data, appropriate variable transformations were applied to fulfill the assumptions of normality or homogeneity of variance before conducting one-way ANOVA. If the assumptions were still not met post-transformation, non-parametric methods were employed for statistical analysis. The Least Significant Difference (LSD) method was used for post hoc pairwise comparisons between experimental and control groups, with a significance threshold of *p* < 0.05 indicating significant differences.

## 3. Results

### 3.1. IR-HepG2 Cell Model Construction

**Figure 1:**
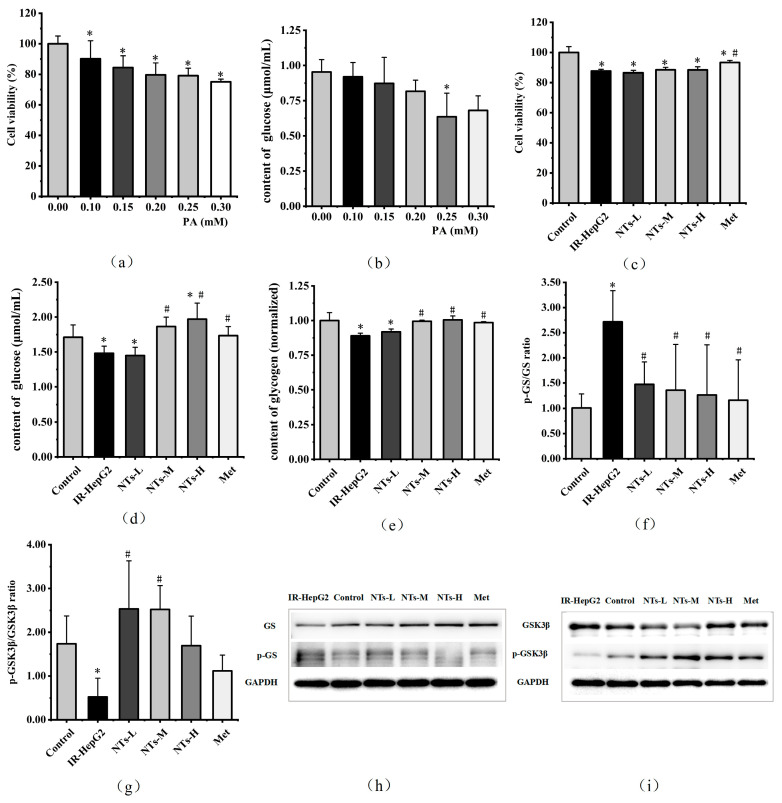
Effects of PA on HepG2 cell viability and glucose consumption and the impact of NTs on the viability, glucose consumption, glycogen content, GS expression, and GSK3β expression in IR-HepG2 cells. (**a**) Cell viability levels in HepG2 cells treated with 0.10–0.30 mM PA; (**b**) glucose consumption levels in HepG2 cells treated with 0.10–0.30 mM PA; (**c**) cell viability levels in IR-HepG2 cells; (**d**) glucose consumption levels in IR-HepG2 cells; (**e**) glycogen content in IR-HepG2 cells; (**f**) Western blot analysis of p-GS/GS in IR-HepG2 cells; (**g**) Western blot analysis of p-GSK3β/GSK3β in IR-HepG2 cells; (**h**) GS and p-GS protein levels were monitored by Western blot analysis; (**i**) GSK3β and p-GSK3β protein levels were monitored by Western blot analysis. * Compared to the blank control group *p* < 0.05, # Compared to the model group *p* < 0.05. GS, glycogen synthase; GSK3β, glycogen synthase kinase.

Palmitic acid elicits lipotoxic effects in cells, leading to a dose-dependent reduction in the viability of HepG2 cells following exposure to different concentrations of PA. According to the cell viability assay data, concentrations of PA equal to or greater than 0.10 mM significantly reduced cell viability compared to the control group (PA concentration of 0), with a statistical significance of (*p* < 0.05). Notably, the cell viability of the 0.25 mM dosage group was approximately 80%. Analysis of glucose consumption revealed that intervention with 0.25 mM PA led to a significant decrease in glucose consumption compared to the control group (*p* < 0.05), indicating the development of insulin resistance under PA induction and subsequent reduction in glucose consumption. Therefore, the PA concentration of 0.25 mM was selected for further investigation in this study. The results are shown in Figure 1a,b.

### 3.2. The Effect of Exogenous NTs on the Viability of IR-HepG2 Cells

The examination of exogenous NTs’ influence on IR-HepG2 cell viability demonstrated a notable decline in cell viability within the model group when contrasted with the control group (*p* < 0.05). Despite exposure to diverse concentrations of exogenous NTs, cell viability failed to reach levels equivalent to those observed in the blank control group (*p* > 0.05). Nevertheless, the Met group administered with metformin demonstrated a partial restoration in cell viability, exhibiting a statistically significant disparity (*p* < 0.05), albeit not reaching parity with the control group. The results are shown in Figure 1c.

### 3.3. The Effect of Exogenous NTs on Glucose Consumption in IR-HepG2 Cells

Insulin resistance precipitates a decline in glucose consumption within IR-HepG2 cells. Relative to the blank control group, the model group evidenced a significant reduction in glucose consumption (*p* < 0.05). Among the cohorts treated with exogenous NTs, the NTs-L dosage group failed to exhibit statistical significance in glucose consumption compared to the model group (*p* > 0.05). However, the NTs-M group exhibited increased glucose consumption compared to the model group, showing a statistically significant difference (*p* < 0.05) and effectively restoring levels comparable to the control group. Similarly, the NTs-H group displayed elevated glucose consumption compared to both the model and blank control groups, with a statistically significant difference (*p* < 0.05). Additionally, the Met group demonstrated heightened glucose consumption relative to the model group, with a statistically significant disparity (*p* < 0.05), effectively restoring levels comparable to the control group. The experimental findings underscore the potential of exogenous NTs to mitigate the decline in glucose consumption induced by insulin resistance. The results are shown in Figure 1d.

### 3.4. The Effect of Exogenous NTs on Glycogen Synthesis in IR-HepG2 Cells

Insulin resistance is associated with the inhibition of hepatic glycogen synthesis [29]. Compared to the blank control, the model group exhibited a significant reduction in cellular glycogen content (*p* < 0.05). However, the NTs-M, NTs-H, and Met groups demonstrated increased glycogen content relative to the model group, with statistically significant differences (*p* < 0.05), effectively restoring levels comparable to the control group. The phosphorylation level of GSK3β in the model group was lower than that in the control group, showing a statistically significant difference (*p* < 0.05). The phosphorylation levels of GSK3β in the NTs-L and NTs-M groups were restored to the level of the control group and surpassed those in the model group, with statistically significant differences (*p* < 0.05). Furthermore, the phosphorylation level of GS in the model group exceeded that in the control group, exhibiting a statistically significant disparity (*p* < 0.05). However, the phosphorylation levels of GS in the NTs-L, NTs-M, NTs-H, and Met groups were reinstated to the level of the control group and were lower than those in the model group, with statistically significant differences (*p* < 0.05). These observations suggest that exogenous NTs enhance glycogen synthesis, leading to an elevation in its content in IR-HepG2 cells. The results are shown in Figure 1e–i.

### 3.5. The Effect of Exogenous NTs on Glycolysis/Gluconeogenesis in IR-HepG2 Cells

**Figure 2:**
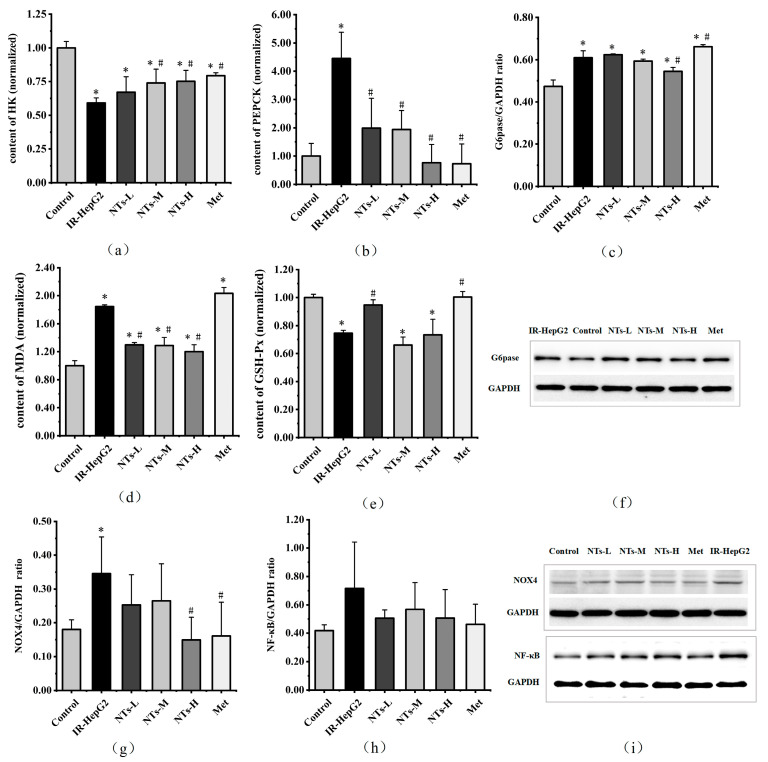
Effects of NTs on the expression of HK, PEPCK, G6pase, MDA, GSH-Px, NOX4, and NF-κB in IR-HepG2 cells. (**a**) HK content in IR-HepG2 cells; (**b**) PEPCK content in IR-HepG2 cells; (**c**) Western blot analysis of G6pase/GAPDH in IR-HepG2 cells; (**d**) MDA content in IR-HepG2 cells; (**e**) GSH-Px content in IR-HepG2 cells; (**f**) G6pase protein level was monitored by Western blot analysis; (**g**) Western blot analysis of NOX4/GAPDH in IR-HepG2 cells; (**h**) Western blot analysis of NF-κB/GAPDH in IR-HepG2 cells; (**i**) NOX4 and NF-κB protein levels were monitored by Western blot analysis. * Compared to the blank control group *p* < 0.05, # Compared to the model group *p* < 0.05. HK, hexokinase; PEPCK, phosphoenolpyruvate carboxykinase; G6pase, glucose-6-phosphatase; MDA, malondialdehyde; GSH-Px, glutathione peroxidase; NOX4, NADPH oxidase 4; NF-κB, nuclear factor-kappa B.

The experimental outcomes revealed a significant elevation in PEPCK content within the model group compared to the blank control group (*p* < 0.05). However, treatment with NTs-L, NTs-M, NTs-H, and Met resulted in a significant reduction in PEPCK content relative to the model group (*p* < 0.05), effectively restoring it to levels comparable to the control group. Notably, HK content in the model group was significantly lower than that in the control group (*p* < 0.05). Although the NTs-L group showed no significant impact on HK content, the NTs-M, NTs-H, and Met groups exhibited higher HK content compared to the model group (*p* < 0.05), albeit not reaching the levels observed in the control group. Furthermore, PA intervention led to a significant increase in G6pase protein expression compared to the control group (*p* < 0.05). Interestingly, G6pase expression in the NTs-H group was lower than that in the model group (*p* < 0.05), effectively restoring it to a level equivalent to the control group, though not reaching the same level as the control group. These findings underscore the efficacy of exogenous NTs in modulating hepatic enzyme expression levels in IR-HepG2 cells. The results are shown in Figure 2a–c,f.

### 3.6. The Effect of Exogenous NTs on Oxidative Stress in IR-HepG2 Cells

Oxidative stress is a well-established contributor to insulin resistance [30]. The results of this study indicate that exposure to palmitic acid (PA) led to a decrease in GSH-Px levels and an increase in MDA levels in HepG2 cells, both of which were statistically significant (*p* < 0.05). Treatment with NTs-L and Met resulted in higher GSH-Px levels compared to the model group, effectively restoring them to levels comparable to the control group (*p* < 0.05). Additionally, MDA levels in the NTs-L, NTs-M, and NTs-H groups were lower than those in the model group, although not fully reaching control group levels (*p* < 0.05). The expression levels of NOX4 protein were found to be elevated in the model group compared to the control group (*p* < 0.05). However, both the NTs-H and Met groups exhibited lower NOX4 protein expression levels compared to the model group, effectively restoring them to levels equivalent to the control group (*p* < 0.05). These findings suggest that exogenous NT intervention can alleviate PA-induced oxidative stress in insulin-resistant HepG2 cells. The results are shown in Figure 2d,e,g,i.

### 3.7. The Effect of Exogenous NTs on NF-κB in IR-HepG2 Cells

The study findings revealed no significant disparity in NF-κB protein expression levels among the groups (*p* > 0.05). However, a comparative analysis unveiled higher NF-κB protein expression in the model group than in the control group. Conversely, NF-κB protein expression levels were notably lower in the NTs-L, NTs-M, NTs-H, and Met groups compared to the model group, suggesting the potential of exogenous NT intervention in reducing NF-κB protein expression levels in insulin-resistant HepG2 cells. The results are shown in Figure 2h,i.

### 3.8. The Effect of Exogenous NTs on Insulin Signaling Pathway Proteins in IR-HepG2 Cells

**Figure 3:**
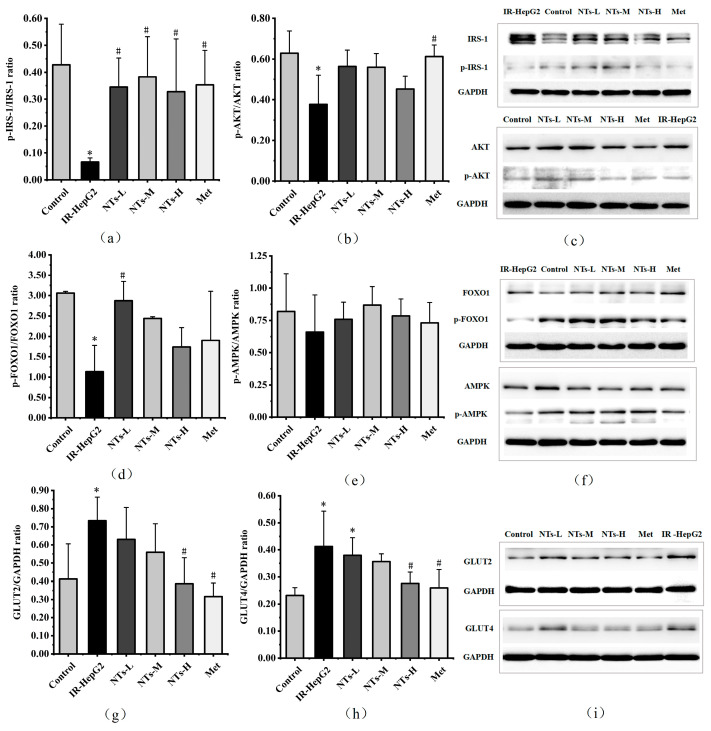
Effects of NTs on the expression of IRS-1, AKT, FOXO1, AMPK, GLUT2, and GLUT4 in IR-HepG2 cells. (**a**) Western blot analysis of p-IRS-1/IRS-1 in IR-HepG2 cells; (**b**) Western blot analysis of p-AKT/AKT in IR-HepG2 cells; (**c**) IRS-1, p-IRS-1, AKT, and p-AKT protein levels were monitored by Western blot analysis; (**d**) Western blot analysis of p-FOXO1/FOXO1 in IR-HepG2 cells; (**e**) Western blot analysis of p-AMPK/AMPK in IR-HepG2 cells; (**f**) FOXO1, p-FOXO1, AMPK, and p-AMPK protein levels were monitored by Western blot analysis; (**g**) Western blot analysis of GLUT2/GAPDH in IR-HepG2 cells; (**h**) Western blot analysis of GLUT4/GAPDH in IR-HepG2 cells; (**i**) GLUT2 and GLUT4 protein levels were monitored by Western blot analysis. * Compared to the blank control group *p* < 0.05, # Compared to the model group *p* < 0.05. IRS-1, insulin receptor substrate-1; AKT, protein kinase B; FOXO1, forkhead box O1; AMPK, AMP-activated protein kinase; GLUT2, glucose transporter 2; GLUT4, glucose transporter 4.

To explore the impact of exogenous NTs, we conducted an analysis of IRS-1, AKT, and FOXO1 protein phosphorylation and total expression levels. In comparison to the control group, IRS-1 phosphorylation levels significantly decreased (*p* < 0.05). However, in the NTs-L, NTs-M, NTs-H, and Met groups, IRS-1 phosphorylation levels rebounded to levels akin to the control group and surpassed those in the model group, with statistically significant disparities (*p* < 0.05). AKT phosphorylation levels were notably lower in the model group compared to the control group, with statistical significance (*p* < 0.05). Although exogenous NTs at varying doses did not induce significant alterations in AKT phosphorylation levels compared to the model group, the data suggested that AKT phosphorylation levels in each dose group generally exceeded those in the model group, exhibiting no statistically significant distinctions compared to the normal group (*p* > 0.05), thereby restoring them to levels akin to the control group. Furthermore, FOXO1 phosphorylation levels were lower in the model group than in the control group, with statistical significance (*p* < 0.05). However, in the NTs-L group, FOXO1 phosphorylation levels surpassed those in the model group, exhibiting statistically significant differences (*p* < 0.05), thereby restoring them to levels akin to the control group. These experimental findings underscore the potential of exogenous NTs in mitigating insulin signaling pathway disruptions in IR-HepG2 cells. The results are shown in Figure 3a–d,f.

### 3.9. The Effect of Exogenous NTs on AMPK Activity in IR-HepG2 Cells

AMPK plays a pivotal role in energy metabolism and represents a promising target for managing type 2 diabetes. Primarily, AMPK regulates hepatic glucose homeostasis by suppressing gluconeogenesis. Experimental findings revealed no statistically significant disparity in AMPK phosphorylation levels across the groups (*p* > 0.05). Nevertheless, the data indicate that the model group exhibited lower AMPK phosphorylation levels compared to the control group, whereas the NTs-L, NTs-M, NTs-H, and Met groups demonstrated elevated levels relative to the model group. These observations suggest the potential of exogenous NT intervention in promoting AMPK activation. The results are shown in Figure 3e,f.

### 3.10. The Effect of Exogenous NTs on Glucose Transporter Proteins in IR-HepG2 Cells

GLUT2 and GLUT4 are pivotal glucose transporters. Experimental evidence revealed a significant upregulation in the protein expression of GLUT2 and GLUT4 in PA-induced HepG2 cells (*p* < 0.05). Nevertheless, in the NTs-H and Met treatment groups, GLUT2 and GLUT4 protein expression levels were notably lower compared to the model group, exhibiting statistically significant differences (*p* < 0.05), thereby restoring them to levels akin to the control group. These findings suggest that exogenous nucleotides have the potential to attenuate the aberrant elevation of GLUT2 and GLUT4 protein expression induced by PA. The results are shown in Figure 3g–i.

## 4. Discussions

**Figure 4:**
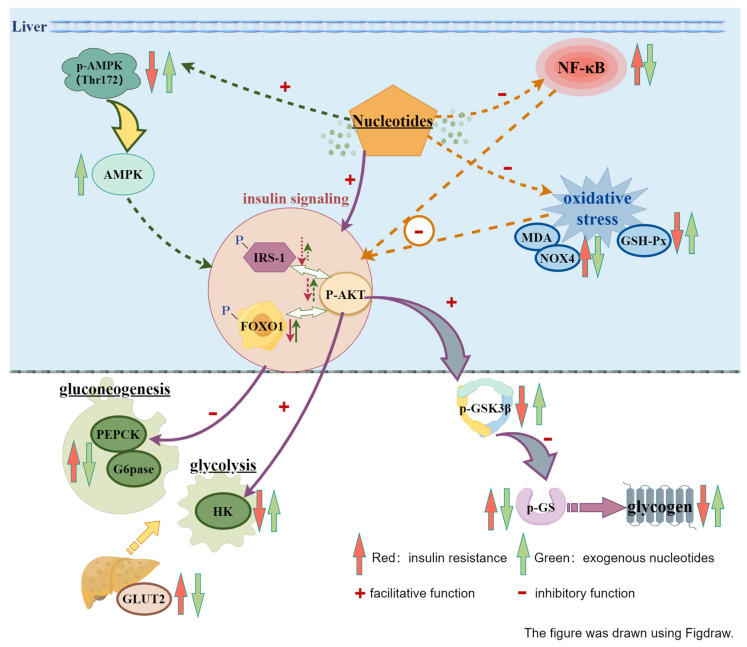
Mechanism of action of exogenous nucleotides on insulin resistance in IR-HepG2 cells.

The results of this study suggest that exogenous NT intervention significantly ameliorates insulin resistance in IR-HepG2 cells. Its mechanism may involve the regulation of AMPK activity, insulin signaling transduction, glycolysis/gluconeogenesis, oxidative stress, GLUT2/GLUT4, and NF-κB expression (Figure 4).

Insulin resistance (IR) often coincides with hyperglycemia. The findings of this study suggest that exogenous NTs could attenuate the glucose consumption induced by PA-induced insulin resistance. AMPK serves as a pivotal regulator in energy metabolism, and its activation in the liver can initiate a cascade of metabolic effects, potentially ameliorating energy metabolism aberrations associated with insulin resistance. AMPK activation necessitates phosphorylation of the activation loop, particularly at the Thr172 site within the α-catalytic subunit kinase domain [31]. In this investigation, exogenous NTs augmented liver AMPK phosphorylation at Thr172 in IR-HepG2 cells, indicating their potential to activate AMPK in the context of insulin resistance. Research has established a close link between impaired AMPK function and insulin resistance as well as diabetes, with its activation shown to mitigate hyperglycemia and enhance insulin sensitivity [32]. Moreover, AMPK exerts significant influence on insulin sensitivity by modulating IRS-1 phosphorylation [33]. Consistent with previous studies [34], a notable reduction in IRS-1 phosphorylated protein expression levels was observed in IR-HepG2 cells, which exogenous NT intervention effectively countered. Additionally, AMPK can activate the PI3K/AKT signaling pathway, crucial in insulin signaling transduction [35]. The intervention with NTs in IR-HepG2 cells elevated the expression levels of phosphorylated proteins of IRS-1, AKT, and FOXO1 in this study, indicative of NTs’ capacity to regulate insulin receptor (IRS-1) expression in IR-HepG2 cells. This further activates the PI3K/AKT pathway and downstream processes, thereby modulating gluconeogenesis, glycolysis, and glycogen synthesis. AKT facilitates glycogen production by phosphorylating and inhibiting GSK3β, a pivotal regulator in hepatic glycogen synthesis. Reduced phosphorylation of GSK3β enhances its activity, thereby elevating glycogen synthase (GS) phosphorylation levels, inhibiting its function, and consequently impeding glycogen synthesis. Activation of the PI3K/AKT pathway in this study likely stimulated GSK3β phosphorylation, diminishing its activity, subsequently lowering GS phosphorylation levels, enhancing GS activity, and ultimately promoting glycogen synthesis. Another essential component in insulin signaling transduction is the FOXO1 protein, a major target of AKT, which has been demonstrated to upregulate the expression of PEPCK and glucose-6-phosphatase. AKT primarily phosphorylates and inhibits the FOXO1 protein, further dampening gluconeogenesis pathways [36]. The activation of the PI3K/AKT pathway in this investigation resulted in heightened levels of phosphorylated FOXO1 protein, thereby suppressing FOXO1 protein expression and further repressing PEPCK and G6pase, consequently inhibiting the gluconeogenesis pathway. This is consistent with the outcomes of NT intervention in IR-HepG2 cells in this study, which demonstrated a suppression of PEPCK and G6pase expression. Additionally, AKT fosters glycolysis and energy production by stimulating the HK enzyme to convert glucose into glucose-6-phosphate. The findings of this study revealed a decrease in HK content in HepG2 cells induced by PA, while exogenous NT intervention counteracted this abnormal decrease in HK, thereby promoting glycolysis in IR-HepG2 cells.

The GLUT family, or Glucose Transporter family, comprises various glucose transporters responsible for glucose transport in different cells and tissues, crucial for maintaining glucose homeostasis. Our study’s results indicated that PA induction significantly increased the protein expression of GLUT2 and GLUT4 in HepG2 liver cells, while exogenous NT intervention reversed this increase, restoring it to normal levels. However, there is inconsistency in the reported protein expression levels of GLUT2 and GLUT4 in liver cells under conditions of insulin resistance. The majority of studies have indicated a reduction in the protein expression levels of GLUT2 and GLUT4 associated with insulin resistance. Intriguingly, our research findings reveal an elevation in the protein expression levels of GLUT2 and GLUT4 in insulin-resistant HepG2 cells. A minority of studies align with our research findings. Sharawy MH et al.’s study demonstrated that a high-fructose diet increased GLUT2 levels in mouse liver [37], inducing insulin resistance in rats, consistent with other literature reports [38,39]. Furthermore, research has shown that aging liver cells exhibit selective insulin resistance, characterized by increased GLUT4 expression [40]. In our investigation, insulin resistance elicited an elevation in the overall protein expression levels of GLUT2 within HepG2 cells. Given the pivotal role of GLUT2 in regulating hepatic glucose equilibrium, this observed augmentation in GLUT2 expression could be construed as a compensatory mechanism in response to the diminished hepatic capacity for glucose uptake and utilization during insulin resistance. Its purpose is to uphold the intricacies of glucose metabolism by expediting glucose uptake and its subsequent metabolic processes, thus counterbalancing the blunting effects on insulin signaling pathways. Conversely, intervention with exogenous NTs resulted in a reduction in GLUT2 protein expression in insulin-resistant HepG2 cells, consequently reinstating glucose metabolism to its normative state. In addition, in the context of insulin resistance, often concurrent with hyperinsulinemia, GLUT4 assumes a pivotal role as an insulin-responsive glucose transporter protein, facilitating glucose uptake through its translocation from the cytoplasm to the plasma membrane. However, within the milieu of insulin resistance, the cellular sensitivity to insulin wanes, resulting in attenuated glucose uptake. To uphold blood glucose homeostasis, compensatory mechanisms may induce an elevation in GLUT4 protein expression levels. Further explanation lies in our detection of total GLUT4 protein levels without distinguishing between cytoplasmic and plasma membrane-bound GLUT4. Prior investigations have delineated that stimulation with palmitic acid prompts a substantial augmentation in cytoplasmic GLUT4 levels within HepG2 cells, prompting the translocation of GLUT4 from the plasma membrane to the cytoplasm, thereby augmenting total GLUT4 abundance [41]. Nevertheless, the subset of GLUT4 responsible for mediating glucose uptake at the plasma membrane undergoes downregulation. In our study’s context, intervention with exogenous nucleotides could potentially facilitate the translocation of GLUT4 from the cytoplasm to the plasma membrane. This process, in turn, may enhance glucose uptake, thereby mitigating insulin resistance and restoring GLUT4 functionality to baseline levels.

The antioxidant properties of exogenous NTs represent a potential mechanism for ameliorating insulin resistance in IR-HepG2 cells. This study revealed that PA induction significantly reduced GSH-Px levels, elevated MDA levels, and upregulated NOX4 protein expression, whereas exogenous NT intervention reversed these deviations. Glutathione peroxidase (GSH-Px), a vital antioxidant enzyme within cells, plays a crucial role in scavenging peroxides, including hydrogen peroxide and organic peroxides. Malondialdehyde (MDA), an organic compound and primary product of lipid peroxidation, serves as a commonly employed biomarker for assessing oxidative stress levels. Moreover, NOX4 has emerged as a pivotal enzyme implicated in oxidative stress, potentially linked to hepatic insulin resistance [42]. The findings of this investigation suggest that exogenous NT administration can mitigate MDA and NOX4 production while enhancing GSH-Px activity in the PA-induced liver insulin resistance model. Additionally, the body of literature indicates that AMPK exerts regulatory control over intracellular oxidative stress levels by inhibiting NADPH oxidase activation and NOX subunit expression, including NOX4, thereby attenuating reactive oxygen species (ROS) generation [43]. Studies by Eid AA and colleagues demonstrated that AMPK activation modulates NOX4 protein expression induced by high glucose levels [44].

This study additionally evaluated the protein expression levels of NF-κB, a transcription factor intricately involved in modulating cellular inflammation and immune responses, particularly relevant in the pathogenesis of insulin resistance, notably in contexts marked by chronic low-grade inflammation. The findings revealed that PA induction escalated NF-κB protein expression levels in hepatic cells, whereas exogenous NT intervention attenuated NF-κB protein expression levels in insulin-resistant hepatic cells. Although additional experimental data are required to validate this conclusion, the existing data nevertheless offer valuable insights. These observations suggest that exogenous NTs might mitigate insulin resistance by modulating inflammatory levels within hepatic cells.

This study aimed to investigate the potential therapeutic effects and underlying mechanisms of exogenous nucleotide mixtures on insulin resistance in hepatic cells. The results demonstrated that exogenous NTs enhanced glucose consumption and facilitated glycogen synthesis in IR-HepG2 cells. This effect primarily involves the synergistic action of multiple cellular signaling pathways. Firstly, exogenous nucleotides may activate AMPK, a crucial cellular energy sensor promoting cellular energy metabolism, thereby influencing the insulin signaling pathway. Secondly, they modulate the phosphorylation level of IRS-1 (insulin receptor substrate-1) and activate the PI3K/AKT pathway, pivotal in the insulin signaling cascade. Additionally, exogenous nucleotides regulate gluconeogenesis and glycolysis processes, thus balancing glucose metabolism pathways within the cell and improving insulin sensitivity. Moreover, they ameliorate oxidative stress status, reducing intracellular oxidative damage levels, a significant factor in insulin resistance development, thereby further enhancing cellular insulin sensitivity. Lastly, exogenous nucleotides potentially diminish intracellular inflammation levels by downregulating inflammatory signaling pathways, indirectly ameliorating insulin resistance.

Overall, the study highlights the synergistic regulation of hepatic insulin resistance by the exogenous nucleotide mixture across multiple pathways, enhancing our understanding of their role in mitigating insulin resistance mechanisms. However, several limitations should be noted. The investigation into the mechanism of action of exogenous nucleotides remains incomplete, lacking precise elucidation of specific upstream and downstream mechanisms. Furthermore, the investigation into mechanisms that indirectly ameliorate insulin resistance, such as the AMPK and inflammatory signaling pathways mentioned previously, lacked sufficient depth. Future research could incorporate techniques such as gene silencing to target key pathways and genes at the cellular level, employing more comprehensive and precise indicators along with broader research methodologies. Conclusively, the intriguing fluctuations detected in the expression levels of GLUT2 and GLUT4 proteins within our study underline the necessity for extended inquiry. Future research endeavors should aim to explore this phenomenon more comprehensively, thereby elucidating the underlying mechanisms and their broader implications. This approach would further illuminate the mechanisms underlying the effects of exogenous nucleotides on insulin resistance, facilitating their translation into practical applications across diverse populations with increased precision.

## 5. Conclusions

In summary, the outcomes of in vitro cell experiments revealed that palmitic acid induced insulin resistance in HepG2 cells, leading to reduced glucose consumption, impaired glycogen synthesis, heightened oxidative stress, and increased inflammation. Conversely, intervention with various doses of exogenous nucleotides (NTs) was observed to mitigate insulin resistance in IR-HepG2 cells. This intervention regulated the IRS-1/AKT/FOXO1 pathways, resulting in enhanced glucose consumption, restored glycogen synthesis, modulation of hepatic enzyme expression levels, and regulation of the glycolysis/gluconeogenesis process. Furthermore, exogenous NTs may enhance insulin sensitivity by mitigating oxidative stress and inflammation while also activating the AMPK pathway.

## Data Availability

All data generated or analyzed during this study are included in this article. Further inquiries can be directed to the corresponding author.

## Abbreviations

AKT	Protein kinase B
AMPK	AMP-activated protein kinase
FOXO1	Forkhead box O1
G6pase	Glucose-6-phosphatase
GLUT2	Glucose transporter 2
GLUT4	Glucose transporter 4
GS	Glycogen synthase
GSK3β	Glycogen synthase kinase
GSH-Px	Glutathione peroxidase
HK	Hexokinase
IR	Insulin resistance
IRS-1	Insulin receptor substrate-1
MDA	Malondialdehyde
Met	Metformin
NF-κB	Nuclear factor-kappa B
NOX4	NADPH oxidase 4
NTs	Nucleotides
PEPCK	Phosphoenolpyruvate carboxykinase
PI3K	Phosphatidylinositol 3-hydroxy kinase
SOD	Superoxide dismutase
